# Elucidating Amendment Resources for Reclaiming Efficacy of Sodic Soils around Abaya and Chamo Lakes, South Ethiopia Rift Valley

**DOI:** 10.3390/toxics12040265

**Published:** 2024-03-31

**Authors:** Azmera Walche, Wassie Haile, Alemayehu Kiflu, Dereje Tsegaye

**Affiliations:** 1 College of Agricultural Sciences, Arba Minch University, Arba Minch P.O. Box 21, Ethiopia; dassdere@yahoo.com; 2 College of Agriculture, Hawassa University, Hawassa P.O. Box 05, Ethiopia; wassiehaile@yahoo.co.uk (W.H.); alemacnchy@gmail.com (A.K.)

**Keywords:** sodic soil, soil properties, arid regions, gypsum, farmyard manure

## Abstract

Background: Sodic soils are harmful to agricultural and natural environments in Ethiopia’s semi-arid and arid regions, leading to soil degradation and reduced productivity. This study investigated how amendment resources could help improve the chemical properties of sodic soils around the Abaya and Chamo Lakes in the South Ethiopia Rift Valley. Methods: A factorial experiment was conducted to study the effects of gypsum (GYP) and farmyard manure (FYM) on sodic soil reclamation. The experiment had four levels of GYP (0, 50, 100, and 150%) and four levels of FYM (0, 10, 20, and 30 tons ha^−1^), with three replications. The pots were incubated for three months and leached for one month, after which soil samples were collected and analyzed for chemical properties. ANOVA was performed to determine the optimal amendment level for sodic soil reclamation. Results: The study found that applying 10 ton FYM ha^−1^ and gypsum at 100% gypsum required (GR) rate resulted in a 99.8% decrease in exchangeable sodium percentages (ESP) compared to untreated composite sodic soil and a 1.31% reduction over the control (GYP 0% + FYM 0 ton ha^−1^). As a result, this leads to a decrease in soil electrical conductivity, exchangeable sodium (Ex. Na), and ESP values. The results were confirmed by the LSD test at 0.05. It is fascinating to see how different treatments can have such a significant impact on soil properties. The prediction models indicate that ESP’s sodic soil treatment effect (R^2^ = 0.95) determines the optimal amendment level for displacing Ex. Na from the exchange site. The best estimator models for ESP using sodic soil treatment levels were ESP = 1.65–0.33 GYP for sole gypsum application and ESP = 1.65–0.33 GYP + 0.28 FYM for combined GYP and FYM application, respectively. Conclusion: The study found that combined GYP and FYM applications reduced ESP to less than 10% in agriculture, but further research is needed to determine their effectiveness at the field level.

## 1. Introduction

A balanced nutrient application is necessary for long-term agricultural production and soil health since plant nutrients are essential to crop productivity [1]. Nutrient availability in the soil is influenced by the physico-chemical characteristics of the soil and management factors [2]. Since salt ions are more prevalent in alkaline soil, crop growth is limited by the availability of nutrients [3]. The higher concentrations of salt cations such as sodium (Na), calcium (Ca), and magnesium (Mg), along with the associated chloride (Cl), sulfates (SO_4_), carbonate (CO_3_), and bicarbonate (HCO_3_) anions, restrict the availability of critical plant nutrients [4]. Sodic soils are a severe problem, particularly in dry and semi-arid areas [5]. Exchangeable sodium percentages (ESP) > 15, an electric conductivity (EC) of 4 dS m^−1^, and a saturation extract sodium adsorption ratio (SAR) lower limit of 13 are all characteristics of sodic soils. Therefore, the fundamental problem in these soils is Na^+^ [6]. In sodic soils with high sodium concentrations and low ECe, this causes dispersion. When the electrolyte concentration drops below the flocculation value of the clay, it leads to clay dispersion [7]. Low levels of salt are present in sodium-affected soils, which also have weak structural stability, low hydraulic conductivities, and low infiltration rates [8]. Due to poor aeration and insufficient water availability, crops produce less due to these bad physical characteristics. Significant soil erosion might also result from low infiltration rates [9].

Soils containing high concentrations of sodium ions or soluble salts are known as salt-affected soils. Interestingly, it was calculated in the late 1970s that the initial global distribution of these soils covered around 1 billion hectares. However, the global distribution has since undergone sporadic adjustments [10]. Saline and sodic soils significantly impact global food security, leading to stunted growth and lower yields [11]. Salinization and sodification make previously productive land unusable for agriculture, decreasing global food production [12]. By 2050, up to 50% of arable land could be affected by salinity. Coastal areas are particularly vulnerable, and climate change-induced sea levels exacerbate salinization [13]. Globally, nearly 2000 ha of agricultural land is lost to production every day because of salinization [14]. In Ethiopia, it was reported that there are over 11 million hectares of unproductive, naturally salt-affected soils, ranking first in Africa, followed by Kenya (8.2 million hectares), Nigeria (5.6 million hectares), and Sudan (4.8 million hectares), respectively [15,16]. The dry and semi-arid agro-ecologies, which constitute about half of Ethiopia’s land area, are considered challenging for crop development due to the high salinity level in the soil and water [17]. Arid and semi-arid regions in the country have soils affected by salt, particularly sodic ones [18]. The impact of saline-sodic and sodic soil on crop production in the irrigated lands of Ethiopia’s arid and semi-arid regions is severe. The presence of sodic soil due to the expansion of irrigated agriculture seriously threatens the sustainability of crop yields in the area [9]. The pH of sodic soils ranges from 8.5 to 10. The hydrolysis of Na_2_CO_3_ is what causes the high pH. Cl, SO_4_, and HCO_3_ are the three main anions in the soil solution of sodic soils, with smaller amounts of CO_3_^2−^. Ca^2+^ and Mg^2+^ precipitate because of the high pH and the presence of CO_3_^2−^, which results in a low soil solution of Ca^2+^ and Mg^2+^ [19]. In addition to Na^+^, K^+^ is another soluble and exchangeable cation that may be present in these soils [20]. Techniques like drip irrigation, precision agriculture, salt-tolerant crops, and gypsum can help manage these problems [21,22]. A multi-pronged approach involving climate change, innovative technologies, and sustainable land management practices is needed to address these challenges [23,24].

According to [25], traditional sodic soil restoration often entails applying and incorporating gypsum into the soil as well as applying extra water for leaching. It is crucial for water to pass through and into the soil. It dissolves the gypsum, makes it easier for calcium to travel to the exchange sites, and gets rid of the sodium that was once exchangeable [26]. This, in addition to applying gypsum (CaSO_4_ 2H_2_O) or CaCl_2_ to remove the exchangeable Na^+^ from the exchange sites, can be an effective way to improve soil quality and promote better plant growth [27]. In the exchange of Ca^2+^ and Na^+^ ions, Na^+^ is leached out as a soluble salt, like Na_2_SO_4_ or NaCl. In addition, CaSO_4_ and CaCl_2_ can also increase permeability by increasing the electrolyte concentration [28]. Extensive research has been undertaken on the properties of sodic soils and their amelioration, with a focus on the physical aspect [29,30]. While using chemical amendments to remove sodium from the soil’s cation exchange sites is required to reclaim sodic soils, leaching is the most efficient way to remove soluble salts from the rhizosphere in salty soils [27,31,32]. Saline-sodic soils can be made productive to yield a good crop through proper management practices [33]. In this regard, finding the most effective reclamation technique or combination of technologies to enhance crop yields and manage farmland in saline-sodic soil is essential [34]. Gypsum is a commonly used amendment material due to its availability and affordability [8]. According to [35], combining farm yard manure (FYM) and gypsum (GYP) can effectively restore sodic soils. Apparently, this process works by increasing the level of Ca^2+^ ions while reducing the amount of Na^+^ ions on the exchange sites. In turn, the excess Na^+^ is removed from below the root zone or via leaching water out of the soil profile [36].

Calcium accumulation on the exchange sites can contribute to better soil aggregation in sodic soil, which in turn can help reduce the soil’s bulk density [37]. Calcium is typically obtained from amendments that have either soluble Ca^2+^ or can dissolve Ca^2+^ upon reacting with soil [38]. Gypsum and organic matter have recently been used for soil reclamation [27]. Gypsum, FYM, and PM can impact soil pH, Na concentration, and the availability of nutrients like N and S for plants. It is good to know that this information is used to manage alkaline soils [3]. The incorporation of rice husk can significantly impact the soil’s electrical conductivity (EC), pH, and sodium adsorption ratio (SAR). It has been observed to decrease these parameters [39]. According to a field trial, cow manure was found to improve the physical properties of soil, while rice husk increased the number of soil pores [40].

Organic matter has several benefits for soil. It can enhance soil structure and aggregation, improve hydraulic conductivity, and increase nutrient levels and cation exchange capacity [41,42]. Adding a combination of organic and inorganic materials to soil can help speed up the process of SOM mineralization. These can, in turn, improve the concentration of plant nutrients in a soil solution. It is also interesting that saline-sodic soils typically require higher organic matter levels to increase production [3]. FYM and gypsum agents can complement each other in several ways, such as improving soil health [3]. According to research, FYM provides organic matter and helps calcium ions move into the soil more easily. This is particularly important for sodic soils, where gypsum alone can slowly remove sodium. However, when FYM is added, the process is accelerated as it improves soil structure and allows for better movement of calcium ions and sodium displacement [26,43].

Combining FYM and gypsum can provide a synergistic approach to soil reclamation and sustainable agricultural practices. The long-term effects of FYM on soil structure and nutrient availability also extend the benefits of gypsum application. It can reduce the need for frequent additions of gypsum and ensure sustained soil improvement, which is beneficial for sustainable farming [44,45]. It is crucial to have site-specific information on soil management to ensure the best possible product for farmers. Investigating sound land management practices for the reclamation of salt-affected soils around Abaya and Chamo Lakes in South Ethiopia Rift Valley is essential, especially considering the long history of agriculture in that region. Finding sustainable solutions is critical to enable farmers to continue their livelihoods while preserving the environment for future generations. This will ensure that site-specific management advice based on site-specific data can be provided to effectively utilize the limited land resources. A thorough soil examination is necessary to ensure it is utilized to its full potential. Using farmyard manure and gypsum together to restore soil quality sounds like an exciting approach that could have positive results. To that end, this experiment successfully improves the soil in the study area. A research study was conducted to replace sodium and lower the exchangeable sodium percentages (ESP) of sodium-affected soil to a level that is suitable for agriculture. In general, the rearrangement lies in combining the individual strengths of gypsum and farmyard manure to achieve a more effective and sustainable approach to reclaiming salt-affected sodic soil. The study utilized multivariate analysis to select the optimal treatment model for reclaiming sodic soil through gypsum and farmyard manure. It aimed to determine and compare the appropriate levels of reclaiming materials required for the process.

## 2. Materials and Methods

### 2.1. Descriptions of the Study Location and Climate

**Figure 1:**
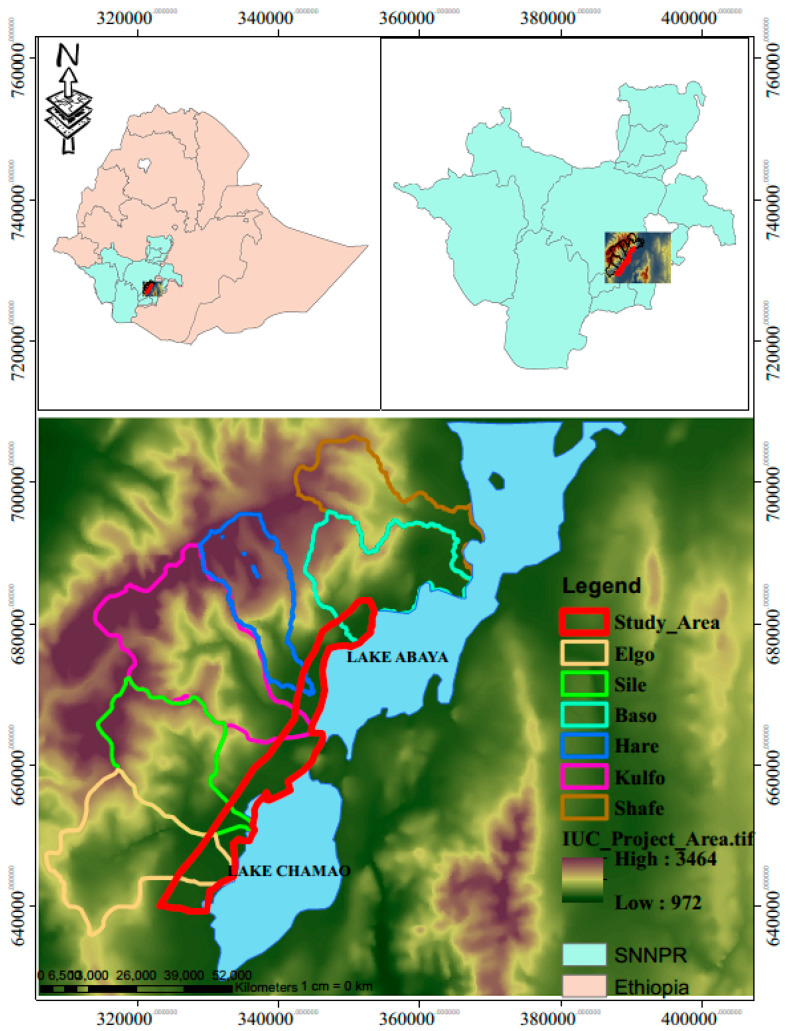
Location map of the project area and the study area.

The Abaya-Chamo drainage basin is a sub-basin of the South Ethiopia Rift Valley that splits Ethiopia down the middle in a north–south direction. The basin comprises two lower-lying lakes, Abaya Lake and Chamo Lake [46,47]. The study region is between 5°50′00″ N and 60°10′0″ N in latitude and between 37°26′0″ E and 37°40′0″ E in longitude, and contains four watersheds—Elgo, Sile, Baso, and Shafe—that cover a total area of 807 km^2^. The Baso and Shafe catchments drain Lake Abaya, while the Elgo and Sile catchments drain Lake Chamo. The main objective of the Arba Minch University (AMU) and Institutional University Cooperation (IUC) Program (Belgium universities ) is to reduce land degradation through sustainable rural land use in the South Ethiopia Rift Valley. AMU-IUC Project 4, VLIR-UOS, near Abaya and Chamo Lakes, was selected for the study due to its accessibility and potential for crop production and sustainable agriculture. The study’s scope covered an area of 2019 sq km (Figure 1).

**Figure 2:**
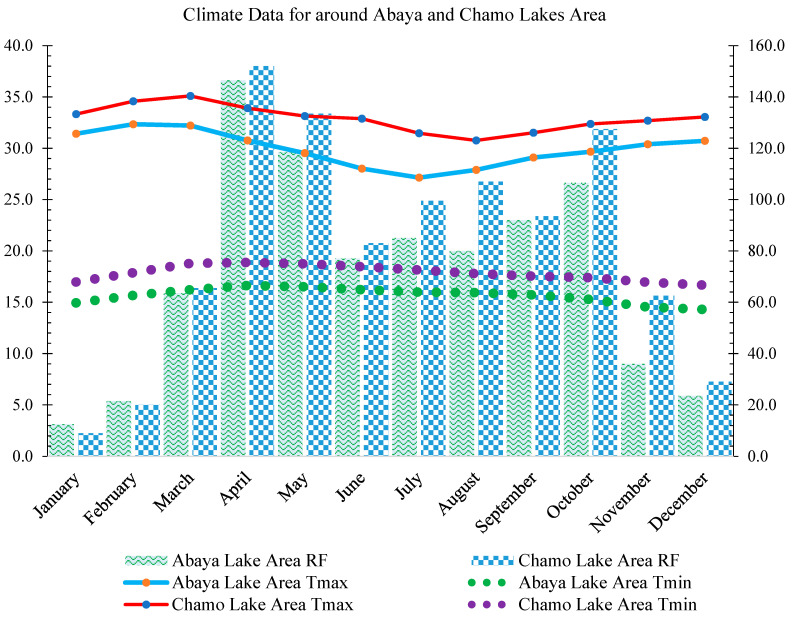
Annual climate data around Abaya and Chamo Lakes in South Ethiopia Rift Valley (1983–2020 average), where rainfall (RF) is in millimeters (mm) and temperature (T) is in degrees Celsius (°C) (Source: AMU-IUC meteorology station).

The climate around the Abaya and Chamo Lakes basin region is tropical, hot, and semi-arid [48]. The bimodal rainfall system in the Abaya-Chamo watersheds seems to be assisted by a humid breeze coming from the Indian Ocean, which is brought in by the inter-tropical convergence zone (ITCZ). Altitude plays a role in the distribution of rainfall. The region experiences short rains in spring (belg) and long rains in summer (kremt), resulting in a bimodal rainfall distribution in most parts of the watershed [49]. In the study area, the rainfall peaks during April and May. On the other hand, the lowest rainfall is recorded during January and February. The temperature is high for three months in the study area. For instance, around Chamo Lake, the temperature increases in February, March, and April, while around Abaya Lakes, the temperature is high during January, February, and March. The mean annual rainfall in the area ranges from 500 to 1100 mm, and the average yearly air temperature is 17–39 °C. According to the AMU-IUC Project 4 (Figure 2), the mean soil temperature ranges from 22 to 35 °C depending on the depth. Agriculture seems to be dominant in the area, with crops such as banana, mango, papaya, maize, cotton, sweet potato, tomato, onion, and haricot beans being cultivated. However, it seems that soil salinity and sodicity are also present in the area. These phenomena are brought on by factors of nature such nearby water tables, weathering rocks and minerals, low rainfall, and high evaporation rates. Unfortunately, these problems are made worse by human actions including inadequate irrigation, deforestation, and overgrazing of livestock [50].

### 2.2. Soil Sampling and Preparation

Collecting bulk soil samples involves using a nursery auger and spades to randomly collect soil from 0–20 cm depth to be reclaimed at a soil depth of identified sodic soil sites around Abaya and Chamo Lakes by Walche et al. [47]. After collection, the soil samples are taken to the lab and spread out on a polythene sheet to dry naturally. The soil samples are carefully combined and sieved through a 5 mm sieve to reduce soil heterogeneity. The laboratory procedures outlined by US Salinity Laboratory Staff are followed during the experiments [51]. The initial soil was analyzed and mentioned for pH, EC, exchangeable bases (Ca^2+^, Mg^2+^, K^+^, and Na^+^), CEC, ESP, and SAR as per Table 1. A pH meter with a combined glass electrode in water (H_2_O) was used to measure the pH of the soil at a ratio of 1:2.5 soil to water, in accordance with [52] recommendations. Saturated soil paste extracts were used to measure electrical conductivity using a conductivity meter, as described by [53]. A pH 7.0 solution of 1 M ammonium acetate (NH_4_OAc) was used to extract the exchangeable bases (Ca^2+^, Mg^2+^, K^+^, and Na^+^) from the soil [54]. Exchangeable Ca^2+^ and Mg^2+^ were determined in the leachate using an atomic absorption spectrophotometer, but exchangeable K^+^ and Na^+^ were determined via flame photometry [55]. From the NH_4_^+^ saturated samples, which were then replaced by K^+^ using a KCl solution, the soil’s potential cation exchange capacity (CEC) was determined. K^+^ displaced ammonium, which was quantified using the micro-Kjeldahl technique, and the excess salt was removed by washing with ethanol [56] and reported as CEC. The sodium adsorption ratio (SAR) was calculated by the procedure outlined in Hand Book No. 60 [57]. ESP were calculated as the percentages of the exchangeable Na to the soil’s CEC.

### 2.3. Laboratory Analysis for Irrigation Water Quality

The initial irrigation water was analyzed as per Table 1. The analysis of the physio-chemical parameters of the samples was carried out using standard laboratory procedures. The pH (H_2_O) and ECw were determined with the help of a pH meter and an electrical conductivity meter, respectively. A flame photometer determined soluble Na^+^ and K^+^ [58], while soluble Ca^2+^ and Mg^2+^ were analyzed directly by an atomic absorption spectrophotometer [59]. Using a technique from [60], the argentometric method was used to measure chloride (Cl^−^), calcium carbonate (CaCO_3_), and bicarbonate (HCO_3_^−^) by a process involving titrating against a silver nitrate standard solution with potassium chromate indicator, while spectrophotometric analysis was used to determine the levels of phosphorous (PO_4_^3−^), nitrate (NO_3_^−^), nitrite (NO_2_^−^), and sulphate (SO_4_^2−^) [61]. The following equation was used to estimate the sodium adsorption ratio (SAR), as recommended by [57]. The ion concentrations in this relationship are given in milligrams per liter or milliequivalents per liter, as stated previously.

(1) $$\mathrm{SAR}=\frac{\mathrm{Na}\;\mathrm{meq}/L}{\sqrt{(\mathrm{Ca}+\mathrm{Mg})/2}}$$

The following formula was used to estimate the residual sodium carbonate (RSC) in irrigation water, as recommended by [62], to see its impact on the soil’s salt content. The concentrations of each ion in this relationship are given in milliequivalents per liter.

(2) $$\mathrm{RSC}=({\mathrm{CO}}_{3}+{\mathrm{HCO}}_{3})\;-\;(\mathrm{Ca}+\mathrm{Mg})$$

### 2.4. Amendments Preparation and Application

Decomposed farmyard manure (FYM) was selected as the organic amendment for this experiment. The organic amendment was ground into a finer form and passed through a 2 mm sieve to ensure uniformity. The soil samples were mixed and crushed for the pot experiment to achieve a uniform dry bulk density of 1.2 g cm^−3^ [63]. It is significant to note that due to variations in soil packing and other reasons, the bulk density of each pot can vary [64]. The procedures used for the study were as stated by [65]. The process outlined involved using agricultural grade gypsum (GYP) powder of 98% purity that was sieved through a 2 mm sieve to ensure uniformity and high solubility. This was then mixed with farmyard manure in the pots at a depth of 20 cm. According to the USSLS (1954) the amounts of added gypsum were determined to reduce ESP to 10%, which is acceptable [51]. We used the methods established by [66] to determine the appropriate amount of gypsum needed to replace exchangeable sodium to achieve the desired level of sodicity for a given unit of land area with sodic soils.

(3) GR=CEC∗d∗BD∗0.81(ESPi−ESPF)/f

where *BD* = bulk density of soil (1.4 g cm^−3^), *GR* = required amount of GYP in (10 t ha^−1^), *CEC* = cation exchange capacity in (52.1 cmol (+)/kg) of soil, *d* = depth (0.2 m) of soil to be reclaimed and soil structure has to be improved, *ESPi* = actual (95%) ESP of the soil as determined by analysis, *ESPf* = final ESP to be ascertained after reclamation (10%), and *f* = purity of gypsum applied (98%).

### 2.5. Experimental Design, Treatments and Laboratory Analysis

Combining gypsum (GYP) and farmyard manure (FYM) for sodic soil reclamation is not based on a single, specific protocol. It draws upon several scientific principles (GYP) and findings from various studies (FYM) with different methodologies. Accordingly, the GYP levels were calculated from the gypsum requirement principal formula (Section 2.4, Equation (3)), and FYM levels drew upon the recommendations of various studies. A Completely Randomized Design (CRD) was used to set up a factorial experiment with four levels of GYP—0% (0 ton ha^−1^), 50% (5 ton ha^−1^), 100% (10 ton ha^−1^), and 150% (15 ton ha^−1^)—four levels of FYM (0, 10, 20, and 30 tons ha^−1^), and three replications. The number of treatments was 16, with three replicates of 48 plastic pots/plot/. A plastic pot with different bottom and top diameters was used for the study. The plastic pot used in this experiment had a perforated bottom with drainage outlets, a 19.2 cm bottom diameter, a 23 cm top diameter, a 19.5 cm depth, and a 6833.5 cm^3^ capacity. A wider top diameter of plastic pot can allow for better drainage and aeration, and excess water can drain out more readily [67]. In addition, the plastic pot had more holes, and holes positioned strategically can help ensure drainage and prevent waterlogging, even with constricted flow lines [68]. After passing through a 5 mm sieve, five kilograms of air-dried soil was placed in each pot with a factorial combination of the treatments. All pots were incubated in a shade house for 90 days (3 months) and rewetted regularly to maintain FC as per US Salinity Laboratory Staff (1954) [69]. After a period of 90 days of incubation, the soil in each plastic pot was leached for 28 days, which is equivalent to one month. To determine the pore volume of water prior to the leaching process, the soil in the plastic pots was saturated with a specific amount of water from the bottom of the pot until water appeared on the top of the soil [70]. The determined 2.5 L of water was applied to each pot, considering evaporation loss of 5% under ideal conditions with low wind, clay, or organic-rich soil, large pots, and slow and deep irrigation [71]. The application was completed through 4 irrigation cycles per treatment with seven (7) day intervals. In total, 10 L of water was applied to each pot uniformly through 4 rounds. Irrigation water from the Kulfo River, was used for leaching. At the end of the incubation and leaching period, soil samples were collected from pots using a soil corer to collect a cylindrical soil sample from the entire pot depth, dried, and analyzed for chemical properties. The study extracted exchangeable bases (Ca^2+^, Mg^2+^, K^+^, and Na^+^) in soil using 1 M ammonium acetate (NH_4_OAc) solution at pH 7.0 [54]. The extracted exchangeable Ca^2+^ and Mg^2+^ in the leachate were determined by an atomic absorption spectrophotometer, while the exchangeable K^+^ and Na^+^ were determined by flame photometry [72]. Using a pH meter and following the methodology outlined by [73], the pH of the soil was determined potentiometrically in the supernatant suspension of a 1:2.5 soil-to-water ratio. Using a conductivity meter, electrical conductivity was determined from a soil saturation extract. The method described in Hand Book No. 60 was then used to calculate the sodium adsorption ratio (SAR) and exchangeable sodium percentage (ESP) [57]. Typically, soil ESP refers to the exchangeable sodium-to-cation exchange capacity ratio. Nevertheless, there are inherent challenges in expressing the link between soluble and exchangeable cations in arid region soils using cation-exchange equations. Despite these challenges, a somewhat empirical approach has been used to successfully relate the relative and total concentrations of soluble cations in the saturation extract of soils to the exchangeable-cation composition [57]. Hence, the ESP formula was developed from the empirical approach used for this study.

(4) $$SAR=\frac{{\mathrm{Na}}^{+}}{{\left(\frac{{\mathrm{Ca}}^{2+}+{\mathrm{Mg}}^{2+}}{2}\right)}^{\frac{1}{2}}}$$

(5) ESP=(100(−0.0126+0.01475(SAR))/1)+(−0.0126+0.01475(SAR))

### 2.6. Data Analysis

Prior to the analysis of variance (ANOVA), the assumption of normality was checked using the Shapiro–Wilk normality test, and two-way ANOVA was used to elucidate the appropriate and best amendment level to reclaim the sodic soil. The LSD test was used to measure the mean separation between treatments and determine the significance at 0.05 SAS, Ver. 9.4 [74].

The model used for sodic soil amendments (GYP + FYM) was as follows:-

Yij=μ+Ai+Bj+ABij+eij

where *Yij* = the soil properties on soil treatments of *i*th GYP and *j*th FYM;

*μ*: overall mean;
*Ai*: the effect of *i*th % (GYP: 0, 50, 100, and 150);
*Bj:* the effect of *j*th *ton/ha* (FYM: 0, 10, 20 and 30);
*ABij*: interaction of the effect of *i*th GYP and *j*th FYM;
e*ij*: error.

Multivariate analyses (PROC COR, PROC REG, PCA, cluster) were carried out to differentiate appropriate or optimum treatment and to develop a best treatment level model to reclaim sodic soil using soil chemical properties with high correlation coefficient (CV %) of treatments with output of soil variables. The impacts of treatments were determined with coefficient of determination (R^2^), C (*p*) statistic, and SE (standard error) across the soil amendments (GYP and FYM). The multiple linear regression models used for fitting uniform soil chemical properties for fixed effects were as follows:$\;A=\pi r^{2}$

$$Y_{j}=\alpha +{\beta_{1}X_{1}+\beta }_{2\;}X_{2\;}+\beta_{3}X_{3\;}+\ldots \ldots \ldots \ldots +\beta_{n}X_{n}+ej$$

where *Y_j_* = dependent variable (soil chemical properties/ESP) $A=\pi r^{2}$;

*α* = intercept;
*X*_1_, *X*_2_, *X*_3_, … *X_n_* = the amendment level (GYP + FYM) used to reclaim sodic soil;
*β*_1_, *β*_2_, *β*_3_, … *βn* = regression coefficient of the independent variables *X*_1_, *X*_2_, *X*_3_, … *X_n_*;
*ej* = residual error.

## 3. Results and Discussion

### 3.1. Initial Soil and Irrigation Water Laboratory Analysis

A preliminary study was conducted to analyze the soil’s physicochemical properties and irrigation water quality before the incubation and leaching experiment. The results of the analysis are presented in Table 1. The soil was found to be heavy clay with a pH of 10.6 and an EC of 3.5 d Sm^−1^. The Exchangeable Sodium Percentage (ESP) was also determined to be 95%, indicating that the soil can be categorized as sodic [10]. According to [75], the availability of most nutrients, pH, and ESP solubility could all be significantly impacted. The irrigation water used for leaching was suitable and safe according to [57] (Table 1).

### 3.2. Effects of Gypsum and Farmyard Manure on Chemical Properties of Sodic Soil under Incubation and Leaching Study

#### 3.2.1. Soil pH and Electrical Conductivity

The study found that the pH of the soil was not substantially (*p* < 0.05) changed by the single or combined application of FYM and gypsum compared to the control. However, the increasing application of gypsum alone decreased the initial pH of the sodic soil, which is quite important for the availability of soil nutrients. Nevertheless, continuous decreases in soil pH could have unintended consequences, affect plant nutrient availability, and alter the soil’s chemical composition undesirably. Hence, it must be followed up with soil pH and maintained (Table 2). This might be the result of applying more gypsum, which would have increased the pace at which the Ca^2+^ and Na^+^ exchange reactions occurred when the concentration of Ca^2+^ rose due to the gypsum dissolving [76]. Combined application of gypsum and FYM had significant effects (*p* < 0.05) on soil EC over the control and other treatments. The highest EC values recorded for the combined applications of gypsum and FYM of gyp0 + 0fym, gyp50 + fym0, and gyp150 + fym0 are 11.84, 11.83, and 11.97 d Sm^−1^, respectively. However, the lowest EC values were recorded for gyp0 + fym30, gyp150 + fym20, and gyp150 + fym150, respectively (Table 3). All the treatments increased the EC value of the initial sodic soil but increased combined application of gypsum and FYM decreased EC observed by FYM over the control, which would require further leaching. This might be because adding gypsum to a soil alters the chemistry of the soil by increasing the quantity of salt that is dissolved, which prevents the clay component from expanding and dispersing. Generally, the study revealed an increase in soil electrical conductivity, indicating potential salt accumulation. While beneficial for reducing sodicity, high electrical conductivity can hinder plant water and nutrient absorption and must be balanced through critical soil EC testing and control [77]. According to [76], both FYM and gypsum are helpful because they help the process by supplying organic acids that break down native calcium carbonate (CaCO_3_) and release additional Ca^2+^ for replacement. Gypsum provides Ca^2+^ to replace Na^+^. Replacing exchangeable Na^+^ ions with Ca^2+^ or H^+^ enhances water infiltration and soil aggregation.

As FYM levels rise, GYP and other salts dissolve in the soil solution, replacing Na^+^ at the soil exchange site. This could explain the notable decrease in EC with rising FYM levels. This aligned with the findings of [78], who reported that applying both organic and inorganic ameliorants together was a better way to lower the EC of the soil than applying treatments alone. Increased soil porosity and hydraulic conductivity from the simultaneous application of FYM and gypsum at varying rates reduced EC, which enhanced the leaching of dissolved salts [79,80,81]. The increased H^+^ of the soil solution as a result of FYM dissolving gypsum and other salts to replace Na^+^ in the soil exchange site may be the cause of the decrease in EC with combined treatments. This was in agreement with the work of [82], who reported that in order to lower the ECe of soil, the combined application of organic and inorganic ameliorants was preferable to the application of treatments alone. In general, adding gypsum and FYM together, letting them incubate for three months, and then leaching them for one month improved the chemical reaction and allowed the sodic soil exchange site to exchange Na^+^ for Ca^2+^. Similarly, by enhancing the physical characteristics of the soil, the FYM-caused removal of excess ions may be the cause of a decrease in EC [83]. It is supported by the results of other authors who also reported a decline in EC as a result of applying gypsum and FYM together at varying rates [35,80].

#### 3.2.2. Soil Exchangeable Cations (Na^+^, Mg^2+^, Ca^2+^, and K^+^)

**Figure 3:**
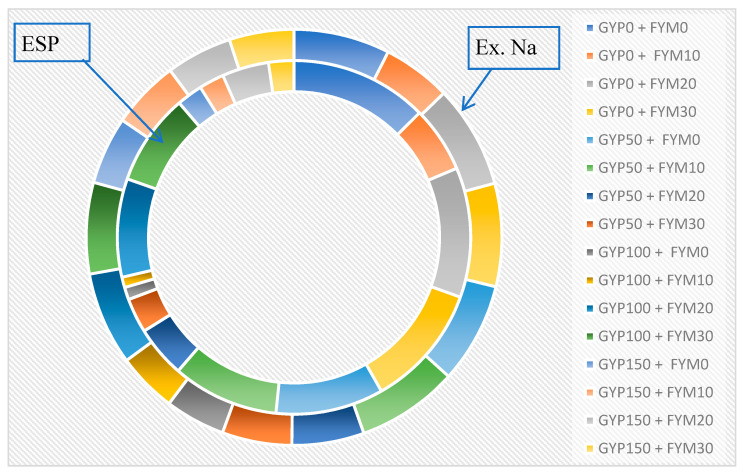
Effect of gypsum (GYP) and farmyard manure (FYM) treatments on exchangeable sodium percentages (ESP) and exchangeable sodium (Ex. Na) of sodic soil under incubation and leaching study. Where GYP 0% would represent no gypsum application (0 tons/hectare, control), GYP 50% would apply half the required amount (5 tons/hectare), GYP 100% would mean using the complete calculated requirement (10 tons/hectare), GYP 150% would apply 50% more than the calculated requirement (15 tons/hectare), FYM 0 tons/hectare would represent no FYM application (control), FYM 10 tons/hectare would apply half the required amount, FYM 20 tons/hectare would mean using the complete calculated requirement, and FYM 30 tons/hectare would apply 50% more than the calculated requirement.

The sole GYP level and FYM level had a significant (*p* < 0.05) effect on the exchangeable Ca^2+^ and K^+^ (Table 2). In contrast, the combined application of gypsum and FYM level and their interactions had more highly significant (*p* < 0.05) effects on exchangeable Na^+^ and Mg^2+^ (Table 3). The amounts of exchangeable Na displaced in response to the applications of the treatments (GYP and FYM) from sodic soil are presented (Table 3). The data in Table 3 demonstrate that the application of treatments at different levels resulted in a significant release of exchangeable Na due to a chemical reaction between the cations in the chemical amendments and the Na in the soil exchange site. It is possible to infer a trend from the figure and table that the concentration of Ca^2+^ increased with the rate increasing of combined application of GYP and FYM, and sole application of FYM had a non-significant effect at *p* < 0.05 while sole application of gypsum had significant effect, and the highest exchangeable calcium (11.69 cmol (+) kg^−1^) was obtained by the application of 150% GYP levels. It is consumed in higher amounts, replacing more Na from the exchange site (Table 3 and Figure 3). However, when treatments were applied in combination rather than independently, exchangeable Na levels were significantly (*p* < 0.05) lower (Table 3). Thus, combined application of gyp150% with 30 t FYM and 20 t FYM ha^−1^ rate reduced exchangeable Na by 2.75% and 2.832.65% over the control, respectively. The application of 10 t FYM ha^−1^ and 100% GR rate of gypsum, followed by 0 t FYM ha^−1^ and 100% GR rate, resulted in a relatively maximum reduction of 95.8% of ex. Na over untreated composite sodic soil and 3.05% of exchangeable Na over the control, respectively (Figure 3). The decrease in exchangeable Na from 4.12 to 2.77 cmol (+) kg^−1^ in this study is likely due to the change in concentration of Ca^2+^ from 7.15 to 11.69 cmol (+) kg^−1^ (Table 3). This is because Ca^2+^ and Na^+^ compete for exchange sites on the soil colloid [84]. When the concentration of Ca^2+^ in the soil solution is increased, more Ca^2+^ will be adsorbed onto the exchange sites, displacing Na^+^ [85]. This approach is beneficial as Na^+^ can have an adverse impact on the soil’s properties and crop growth [20,86]. High levels of exchangeable Na can lead to soil dispersion, reducing water infiltration and aeration. Exchangeable Na can also be toxic to plants, especially at high concentrations [9,87,88]. The study results suggest that increased Ca^2+^ concentration effectively decreases exchangeable Na in the soil [89,90]. This can have several positive benefits for soil health and crop production [91].

The combined application of gypsum and farmyard manure (fym) has a synergistic effect on exchangeable Mg, meaning that the combined effect is greater than the sum of the individual effects (Table 3). This is likely due to several factors, including gypsum displacing exchangeable Na from the soil colloid, allowing more Mg to be adsorbed. FYM improves the soil structure and increases the cation exchange capacity (CEC) of the soil, which provides more binding sites for Mg [92]. Moreover, this increase in CEC, Mg, Ca, and K may benefit plant growth and production. However, excessive levels could lead to plant nutritional imbalances and must be appropriately managed. Farmyard manure (FYM) also contains Mg, which contributes to the increase in exchangeable Mg [93]. Results revealed that exchangeable Mg was increased from 4.29 to 10.1 cmolc·kg^−1^. The highest exchangeable Mg was recorded at the combined gypsum 50% + 30 t FYM ha^−1^ application rate (Table 3). This rate of gypsum is likely sufficient to displace a significant amount of exchangeable Na from the soil colloid [94]. At the same time, the FYM provides additional Mg and improves the soil structure and CEC [95]. Sole application of gypsum increases the amount of calcium in the soil from 7.15 to 11.69 cmol (+) kg^−1^ (Table 2). This could be because gypsum is a calcium sulfate mineral, and when it dissolves in water, it releases calcium ions (Ca^2+^) into the soil solution. These calcium ions can then be adsorbed onto the soil colloid, increasing the amount of exchangeable calcium in the soil [38,96]. Sole application of farmyard manure (FYM) had a significant effect (*p* < 0.05) on potassium (K) in sodic soil by improving soil structure and increasing cation exchange capacity (Table 2). FYM contains K, making it more available to plants [97]. The amount of K in FYM varies depending on the manure source and management practices [98].

#### 3.2.3. Soil Exchangeable Sodium Percentage (ESP)

Combining treatments of 10 t FYM ha^−1^ and gypsum at 100% GR rate, followed by 0 t FYM ha^−1^ and gypsum at 100% GR rate, resulted in a maximum 99.8% drop in ESP over untreated composite sodic soil and a 1.31% decrease over the control, respectively (Table 3 and Figure 3). According to [99,100], sodic soil reclamation benefits from the combined application of FYM and GYP. The ESP was reduced to levels below the allowable limit (ESP < 10%) by the combined application of FYM and gypsum (Table 3 and Figure 3). However, it is crucial to note that even low ESP levels can pose problems, particularly in agricultural soils. Maintaining ESP within safe limits is essential for soil health and crop productivity [101]. The control decreased the ESP to values less than the permissible limit. This could be because the control treatment had a 3-month incubation period and a 1-month leaching time. This created an opportunity for natural sodic soil reclamation by dissolving native calcium carbonate and making calcium available in the solution, thereby reducing exchangeable sodium (Table 3). Dissolving calcium cations through amendment and leaching decreases the soil water’s relative Na^+^ concentration, lowering the exchange complex’s sodicity levels [90,102]. The sulfur will oxidize in the damp soil with the help of soil microbes to generate sulfuric acid, which will dissolve the lime (calcium carbonate) and release its calcium into the solution to replace the sodium on the soil exchange sites [103,104].

The maximal amount of Ca^2+^ that can be dissolved in the incubated and leaching-treated sodic soil with combined gypsum and farmyard manure stays dissolved once applied to the soil. The optimal solution for the reclamation problem will be determined using this quantity as a strong control limitation. After a specific calcium amelioration method, the soil solution’s relative Na^+^ concentration reduced to roughly 47% while the electrolyte content increased, bringing the sodicity in the exchange complex to ESP = 10 [25,105]. Processes related to salinity and sodicity occur over significantly longer periods, from weeks to months. We assume the exchange mechanism is at a local thermodynamic equilibrium since determining the sodium cation in the soil solution and the sodium cation in the exchange complex [106] requires the application of the well-known Gapon equation [102]. This was in agreement with the study of [107,108], who noted that a significant amount of exchangeable Na was released because of the chemical substitution between the cations in the treatments and the Na^+^ in the soil exchange site. The explanation for this could be that gypsum provides soluble Ca^2+^ directly to replace exchangeable Na. At the same time, FYM uses chemical and biological processes to convert the comparatively insoluble carbonates of Ca and Mg that are frequently found in soils into soluble forms that can replace Na^+^ [26,82,109].

### 3.3. Soil Color Change

**Figure 4:**
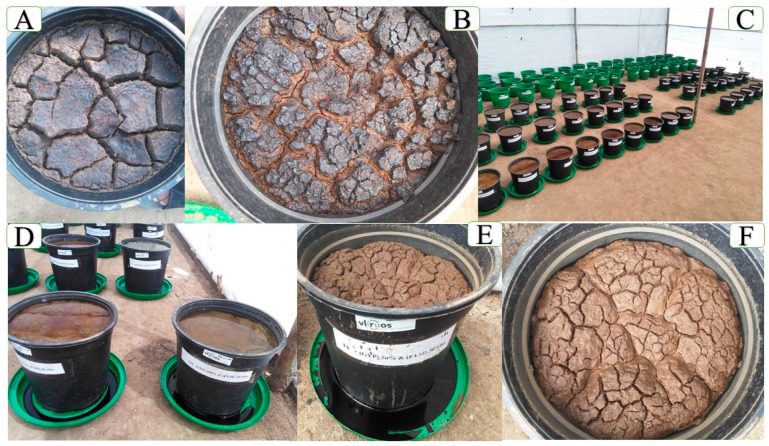
Initial soil for the incubation and leaching study in dark black color (**A**); incubated soil for three months (90 days) (**B**); experiment set up by Completely Randomized Design (CRD) (**C**); leaching incubated sodic soil for one month (28 days) (**D**); reclaimed sodic soil in light brown color at the top and leachate in dark black color at the bottom of the pot concerning last completed leaching pore volume water (**E**); reclaimed sodic soil after three months incubation and the one-month leaching study (**F**).

The color change from dark black to brown in soil after gypsum and farmyard manure application to salt-affected sodic soils can be attributed to several factors [110]. These include the displacement of sodium by calcium, oxidation of organic matter, chemical reactions with iron, and leaching of salts [111,112]. GYP (CaSO_4_) introduces Ca^2+^ ions into the soil. These Ca^2+^ ions replace Na^+^ ions adsorbed onto clay particles, a process called cation exchange. Na^+^ dominance disrupts soil structure and contributes to the dark black color. Replacing Na^+^ with Ca^2+^ improves soil aggregation and drainage, leading to a lighter brown color [27,113,114]. Under sodic conditions, organic matter accumulates and remains un-decomposed, contributing to the dark color. The improved drainage and aeration facilitated by GYP and FYM application can stimulate microbial activity, leading to faster decomposition of organic matter [110,115]. This decomposition releases CO_2_ and dark humic substances, resulting in a lighter brown color. Sodic soils often have reduced Fe, contributing to the dark color. Adding GYP can lead to oxidation of Fe from Fe (II) to Fe (III) oxides. These Fe (III) oxides have reddish-brown hues, which can blend with the remaining organic matter to create a browner color [82,116]. GYP, FYM, and improved drainage can facilitate the leaching of soluble salts from the soil depth (profile). These salts can mask the true color of the soil by appearing as a white crust on the surface. The underlying brown color becomes more prominent as the salts are leached away (Figure 4) [117,118]. Reclaimed sodic soil improves oxygen access to plant roots, loosens up, and lowers in pH, resulting in a brown color [119]. This indicates soil health improvement and a shift from dark black to brown, indicating soil health [120].

### 3.4. Multivariate Analysis of Combined Application of Gypsum and Farmyard Manure Effect on Sodic Soil Chemical Properties under Incubation and Leaching Study

#### 3.4.1. Correlation between Chemical Properties of Reclaimed Sodic Soil

The correlations between the chemical parameters of reclaimed sodic soil are shown in Table 4 by a Pearson correlation matrix. There is a correlation between the soil characteristics and other properties. The exchangeable sodium percentage (ESP) had a strongly positive significant correlation at *p* ≤ 0.01 with soil exchangeable Na (r = 0.95) while it had a strongly negative significant correlation with exchangeable Ca (r = −0.68). Exchangeable Mg showed a significant positive correlation at *p* ≤ 0.01 with exchangeable K (r = 0.63). On the other hand, exchangeable Ca was negatively correlated with soil pH. It showed that combined GYP + FYM amendments had a significant effect on the chemical properties of sodic soils.

#### 3.4.2. Prediction of Exchangeable Sodium Percentages (ESP)

**Figure 5:**
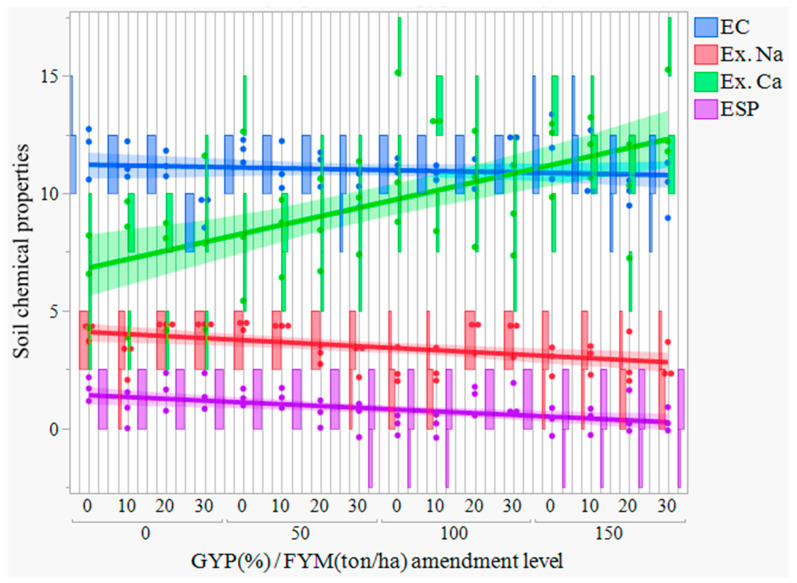
Relationships between the combined application of gypsum (GYP) and farmyard manure (FYM) treatment levels on chemical properties of sodic soil under incubation and leaching study, where EC = electrical conductivity, Ex. Na = exchangeable sodium, Ex. Ca = exchangeable calcium, ESP = exchangeable sodium percentage, GYP 0% would represent no gypsum application (0 tons/hectare, control), GYP 50% would apply half the required amount (5 tons/hectare), GYP 100% would mean using the complete calculated requirement (10 tons/hectare), GYP 150% would apply 50% more than the calculated requirement (15 tons/hectare), FYM 0 tons/hectare would represent no FYM application (control), FYM 10 tons/hectare would apply half the required amount, FYM 20 tons/hectare would mean using the complete calculated requirement, and FYM 30 tons/hectare would apply 50% more than the calculated requirement.

Prediction of soil exchangeable sodium percentage (ESP) for maintaining quality of soil chemical properties show differences among the treatments depending on different levels of amendment contributions, and coefficients of determination in the regression models are indicated in Table 5. The study shows that the sodic soil treatment effect of ESP (R^2^ = 0.95) alone determines the optimum treatment level required to displace exchangeable sodium from the exchange site. The best estimator models for ESP using treatment level for sodic soil were ESP = 1.65–0.33 gyp for sole application of gypsum and ESP = 1.65–0.33 gyp + 0.28 FYM for combined application of gypsum with farmyard manure, represented by gyp100% and gyp100% + fym10 ton/ha treatment levels, respectively (Table 5). Moreover, high dimensional variations were observed among the treatments. Consistently, our study identified negative linear relationships between the combined applied gypsum with farmyard manure levels and ESP, exchangeable Na^+^, and EC. In contrast, positive relationships were observed between gypsum (GYP) plus farmyard manure (FYM) levels and exchangeable Ca^2+^ (Figure 5). Applying gypsum and farm yard manure together could have several beneficial effects on soil, including reducing ESP, exchangeable Na^+^, and EC and increasing exchangeable Ca^2+^ [121,122,123]. These effects are likely because gypsum is a source of calcium, which can help to displace sodium from the soil exchange complex [123]. Farmyard manure is a source of organic matter, which can improve soil structure and increase the cation exchange capacity (CEC) of the soil [92,124,125]. A higher CEC means that the soil can hold more cations, such as calcium, which can help to improve soil nutrient availability and reduce leaching [126].

#### 3.4.3. Principal Components Analysis (PCA) of Reclaimed Sodic Soil Chemical Properties Concerning Different Gypsum and Farmyard Manure Treatments

**Figure 6:**
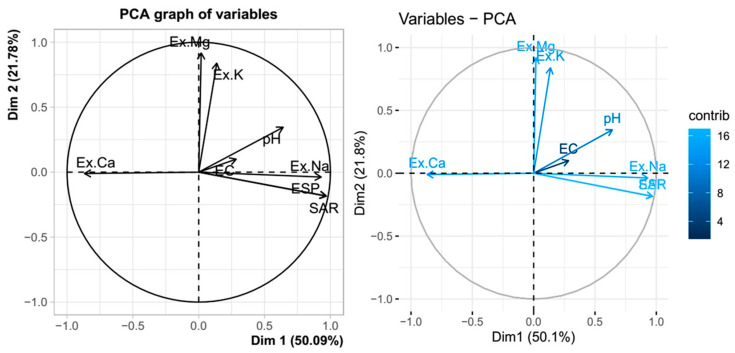
Plot of the chemical characteristics of the reclaimed sodic soil using principal component analysis (PCA) concerning various treatments of farmyard waste and gypsum under leaching and incubation. In this case, pH stands for soil reaction, EC for electrical conductivity, Ex. Na for exchangeable sodium, Ex. Ca for exchangeable calcium, Ex. Mg for exchangeable magnesium, Ex. K for exchangeable potassium, SAR for sodium adsorption ratio, and ESP for exchangeable sodium percentage.

PCA has been used in a number of studies in the literature to assess sodic soils and reclaimed sodic soils [127,128], and the findings of this study show that relationships between the investigated variables of the reclaimed sodic soil and each combination amendment (GYP + FYM) could be established. Figure 6 shows the results of the principal components (PCs) analysis of the combined application of gypsum and FYM on the chemical properties of reclaimed sodic soil. The results showed three main principal components (PCs) with eigenvalues greater than 1 which were considered; the other PCs were neglected. These three PCs explained 86.25% of the studied soil chemical properties’ variability: 50.09%, 21.78%, and 14.38% for PC1, PC2, and PC3, respectively. The first principal component (PC1) represented 50.09% of the total variance of the data and showed positive correlations with the following soil variables: ESP, SAR, Ex. Na, and pH. These ESP, SAR, and Ex. Na soil variables were essential contributions, shown in bold in Table 6 and light blue in Figure 6, to PC1. However, negative correlations and essential contributions were verified for the exchangeable Ca variables, shown in bold in Table 6 and light blue in Figure 6 to PC1. Positive correlations and essential contributions were observed for Ex. K and Ex. Mg, with the second component (PC2) explaining 21.78% of the data variation in the exchange site, while PC3 explained 14.38%; positive correlations were observed for pH and EC (Figure 6 and Table 6). The PCA biplot also revealed that exchangeable Na, SAR, and ESP were strongly positively correlated to each other, while the less strong correlations to EC, pH, Ex. K, and Ex Mg were strongly negatively correlated to Ex.Ca (Figure 6 and Table 6). The variations in the exchangeable complex, soil composition, and soil solution that are confirmed in each treatment are correlated with the changes in the correlation patterns for PC1, PC2, and PC3.

#### 3.4.4. Hierarchical Cluster Analysis of Reclaimed Sodic Soils’ Chemical Properties Concerning Different Gypsum and Farmyard Manure Treatments

**Figure 7:**
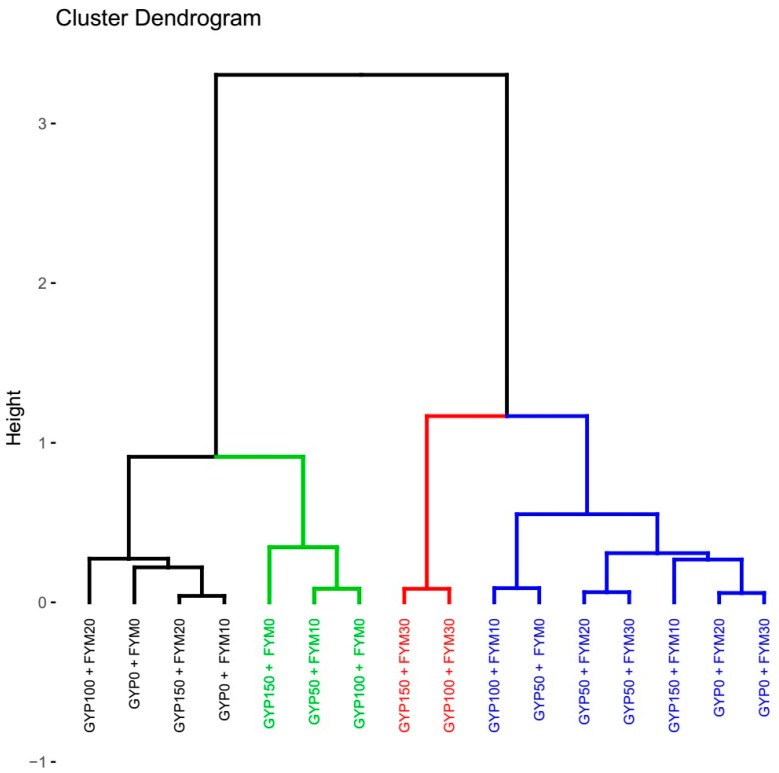
Cluster dendrogram (Ward’s method) of reclaimed sodic soil chemical properties concerning different gypsum and farmyard manure treatments under incubation and leaching study. Where: different color lines have noted that assuming the treatment levels and the reclaimed soils within a cluster have similar properties, they require similar application of the treatment levels and the same management.

In order to determine the similarities and differences in the chemical characteristics of the analyzed reclaimed sodic soil, the current study used cluster analysis over a standardized dataset. Figure 7’s dendrogram, produced by agglomerative hierarchical cluster analysis, shows the differences between four groups of the investigated reclaimed sodic soil based on soil chemical characteristics due to the combined application of gypsum and FYM (sodic variables–pH, EC, Ex. Na, Ex. Ca, ESP, and SAR) of the investigated reclaimed sodic soils. Utilizing multivariate numerical techniques and the R program, soil chemical characteristics were investigated on the reclaimed sodic soil surrounding Abaya and Chamo Lakes. The datasets on modified sodic soil chemical parameters about various combined amendment amounts were subjected to hierarchical cluster analysis. Four clusters were identified by the hierarchical cluster analysis of reclaimed sodic soil’s distance from soil chemical characteristics. The cluster colored black had gyp100 + fym20, gyp0 + fym0, gyp150 + fym20, and gyp0 + fym10 categorized as one cluster. The green-colored luster was clustered as second, including gyp50 + fym0, gyp50 + fym10, and gyp100 + fym0. The third cluster in red represents gyp50 + fym30, and gyp100 + fym30, which are categorized as one cluster. The remaining blue were gathered together in a single group. We note that assuming the treatment levels and the reclaimed soils within a cluster have similar properties, they require similar application of the treatment levels and the same management.

#### 3.4.5. K-Means Clustering of Reclaimed Sodic Soils’ Chemical Properties Concerning Different Gypsum and Farmyard Manure Treatments

**Figure 8:**
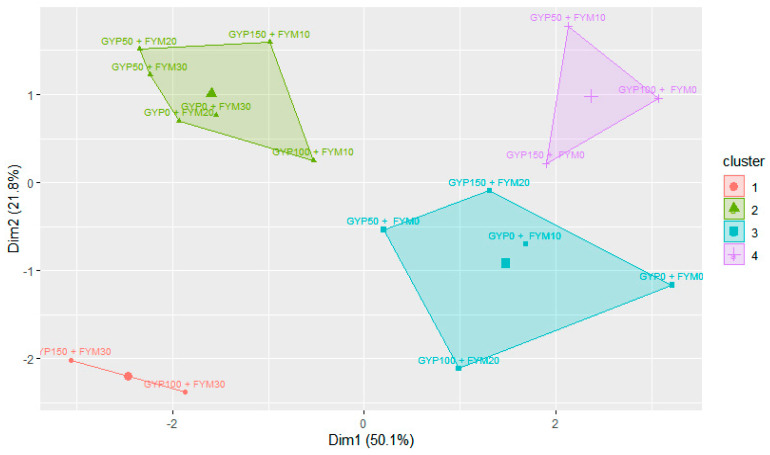
K-means cluster of reclaimed sodic soil chemical properties concerning different gypsum (GYP) and farmyard manure (FYM) treatments under incubation and leaching study.

The K-means cluster analysis based on the combined amendments separated the variance of treatments on the first principal component (PC1) and second principal component, explaining 50.09% and 21.78%, respectively (Figure 8). The combined treatments from cluster 1 in red and cluster 2 in green were located on the left bottom and top side of the PCA plane, respectively. Cluster 3, in blue, and cluster 4, in purple, treatments were in the right bottom and top of the PCA plane and did not differ much.

## 4. Conclusions

Sodic soils are a challenging problem in dry and semi-arid areas, causing soil degradation, productivity decline, and negative impacts on agricultural and natural environments. Adding agricultural-grade gypsum and farmyard manure can increase soil calcium concentration and displace the sodium concentration in sodic soils. In this experiment, different amounts of gypsum and farmyard manure were added to sodic soil to examine the interaction’s effects on pH, exchangeable sodium percentage, electric conductivity, and exchangeable sodium. The findings indicate that the combined application of gypsum and farmyard manure considerably impacted the chemical characteristics of sodic soils. Applying 10 ton FYM ha^−1^ (organic source) along with 100% (10 ton GYP ha^−1^) of gypsum (a chemical amendment) simultaneously can lead to better outcomes. The electrical conductivity of the soil and the percentage of exchangeable sodium declined after three months of incubation and leaching. According to the study, a combined application rate can reduce the sodium content and increase the amount of plant nutrients (Ca, Mg, and K) in the soil. The best estimator models to reduce ESP for the sodic soil using amendment level were ESP = 1.65–0.33 GYP for sole application of gypsum and ESP = 1.65–0.33 GYP + 0.28 FYM for combined application of gypsum with farmyard manure. Agglomerative hierarchical and K-means cluster analysis notes that assuming the treatment levels and the reclaimed soils within a cluster have similar properties, they require similar application of the treatment levels and the same management. Based on research findings, farmyard manure and an appropriate amount of gypsum can assist with managing sodic soils while supporting sustainable crop production. However, further study on changes in microorganisms after treatments and cost analysis will be considered for future research work.

## Figures and Tables

**Table 1:** Selected properties of the untreated composite sodic soil (0–20cm depth) and irrigation water.

Soil			Irrigation Water		
Parameter	Units	Value	Parameter	Units	Value
Texture	---	Heavy Clay	pH	-----	8.3
Clay	%	64	ECw	dS m^−1^	1.184
Silt	%	30	Na^+^	mg L^−1^	17.96
Sand	%	6	K^+^	mg L^−1^	3.90
Bulk Density	gcm^−3^	1.4	Ca^2+^	mg L^−1^	26.20
Gypsum Requirement	tons ha^−1^	10	Mg^2+^	mg L^−1^	13.56
Farmyard Manure	tons ha^−1^	20	Cl^−^	meq L^−1^	0.45
pH	-----	10.6	CO_3_^2−^	meq L^−1^	Nil
EC	dSm^−1^	3.5	HCO_3_^−^	meq L^−1^	2.46
Ex. Na	cmol(+) kg^−1^	48	PO_4_^3−^	meq L^−1^	Nil
Ex. K	cmol(+) kg^−1^	1.16	NO_3_^−^	meq L^−1^	1
Ex. Ca	cmol(+) kg^−1^	3.19	NO_2_^−^	meq L^−1^	1
Ex. Mg	cmol(+) kg^−1^	2.18	SO_4_^2−^	meq L^−1^	0.46
CEC	cmol(+) kg^−1^	52.1	Salinity	% (ppt)	0.59
ESP	%	95	SAR	----	1.04
SAR	---	37.1	RSC	meq L^−1^	0.02

where ECw = electrical conductivity of water; Ex = exchangeable; SAR = sodium adsorption ratio; ESP = exchangeable sodium percentage; RSC = residual sodium carbonate.

**Table 2:** Main effects of gypsum and farmyard manure on sodic soil chemical properties.

GYP (%)				FYM t ha^−1^			
Treatments	pH	Ex. Ca	Ex. K	Treatments	PH	Ex. Ca	Ex. K
0	9.86	7.15 ^c^	0.6	0	9.86	9.59	0.52 ^b^
50	9.81	8.79 ^b^	0.6	10	9.77	9.79	0.68 ^a^
100	9.86	10.64 ^a^	0.55	20	9.78	8.96	0.52 ^b^
150	9.65	11.69 ^a^	0.53	30	9.77	9.94	0.56 ^b^
LSD (0.05)	ns	1.31	ns	LSD (0.05)	ns	ns	0.09
CV%	2.6	24	23	CV%	2.7	30	20

Means within a column followed by the same letters are not significantly different at 0.05 levels.

**Table 3:** Combined application of gypsum and farmyard manure interaction effects on sodic soil chemical properties.

GYP (%)	FYM (t ha^−1^)	EC	Ex. Na	Ex. Mg	SAR	ESP
0	0	11.84 ^ab^	4.12 ^ab^	4.29 ^cd^	1.81 ^a^	1.66 ^a^
	10	11.32 ^abc^	2.94 ^cde^	4.13 ^cd^	1.28 ^bcdef^	0.80 ^bcdef^
	20	11.24 ^abc^	4.41 ^a^	8.15 ^ab^	1.75 ^ab^	1.57 ^ab^
	30	9.33 ^d^	4.42 ^a^	7.72 ^ab^	1.70 ^ab^	1.49 ^ab^
50	0	11.83 ^a^	4.32 ^a^	6.73 ^b^	1.59 ^abc^	1.31 ^abc^
	10	11.09 ^ab^	4.35 ^a^	7.88 ^ab^	1.58 ^abc^	1.29 ^abc^
	20	11.12 ^abc^	3.10 ^bcde^	7.24 ^b^	1.17 ^cdef^	0.63 ^cdef^
	30	10.18 ^bcd^	2.99 ^cd^	10.11 ^a^	1.04 ^def^	0.42 ^def^
100	0	11.14 ^abc^	2.58 ^e^	6.84 ^b^	0.87 ^f^	0.15 ^f^
	10	10.79 ^abcd^	2.56 ^e^	7.99 ^ab^	0.86 ^f^	0.13 ^f^
	20	10.84 ^abcd^	4.00 ^abc^	3.85 ^d^	1.52 ^abcd^	1.19 ^abcd^
	30	11.99 ^a^	3.91 ^abcd^	6.12 ^bc^	1.47 ^abcde^	1.11 ^abcde^
150	0	11.97 ^a^	2.90 ^d^	8.10 ^ab^	0.97 ^ef^	0.31 ^ef^
	10	10.97 ^abc^	2.93 ^cde^	7.96 ^ab^	0.97 ^ef^	0.30 ^ef^
	20	10.05 ^cd^	2.83 ^de^	3.42 ^d^	1.14 ^cdef^	0.57 ^cdef^
	30	10.25 ^bcd^	2.77 ^e^	3.48 ^d^	0.97 ^ef^	0.31 ^ef^
LSD (0.05)		1.42	0.97	2.18	0.46	0.74
CV (%)		7.26	16.91	20	21.16	53.75

Means within a column followed by the same letters are not significantly different at 0.05 levels.

**Table 4:** Pearson correlation matrix among soil chemical properties of reclaimed sodic soils.

	pH	EC	Ex. Na	Ex. Ca	Ex. Mg	Ex. K	ESP
pH	1.00						
EC	0.46	1.00					
Ex. Na	0.47	0.14	1.00				
Ex. Ca	−0.49	−0.16	−0.68 **	1.00			
Ex. Mg	0.30	−0.01	0.05	0.00	1.00		
Ex. K	0.15	−0.02	0.12	−0.18	0.63 **	1.00	
ESP	0.47	0.15	0.95 **	−0.82 **	−0.13	0.03	1.00

** Correlation is significant at the 0.01 level.

**Table 5:** The regression models used for the prediction of soil amendment level from highly correlated soil chemical properties/variable (ESP, Ex. Na).

Treatment (Gyp% + FYM t/ha)	Model	I(α) β_1_X_1_ β_2_X_2_	R^2^	F Value	Pr > F
Gyp100 + FYM10	ESP	1.65–0.33GYP	0.255	15.78	0.000
Gyp100 + FYM10	ESP	1.58–0.33GYP + 0.28FYM	0.257	7.79	0.001
Gyp100 + FYM10	Ex. Na	4.387–0.377GYP	0.234	14.09	0.000
Gyp100 + FYM10	Ex. Na	4.387–0.377GYP + 0.052FYM	0.239	7.0	0.002
Gyp100 + FYM10	Ex.Ca	5.69 + 1.55GYP	0.038	28.33	0.000
Gyp100 + FYM10	Ex. Ca	5.65 + 1.55GYP + 0.020FYM	0.038	13.8	0.000

**Table 6:** Principal component analysis (PCA) of reclaimed sodic soils chemical properties concerning different gypsum and farmyard manure treatments.

PCA	Loading Matrix			PCA	Formatted Loading Matrix		
	Prin1	Prin2	Prin3		Prin1	Prin2	Prin3
Eigenvalue	4.01	1.74	1.15	Eigenvalue	4.01	1.74	1.15
Variance (%)	50.09	21.78	14.38	Variance (%)	50.09	21.78	14.38
Cumulative variance (%)	50.09	71.87	86.25	Cumulative variance (%)	50.09	71.87	86.25
pH	0.64	0.34	0.48	ESP	**0.97**	−0.18	−0.14
EC	0.29	0.10	0.88	SAR	**0.97**	−0.18	−0.14
Ex. Na	0.93	−0.04	−0.17	Ex. Na	0.93	−0.04	−0.17
Ex. Ca	−0.87	0.00	0.10	pH	0.64	0.34	0.48
Ex. Mg	0.02	0.92	−0.09	Ex. Mg	0.02	0.92	−0.09
Ex. K	0.15	0.83	−0.27	Ex. K	0.15	0.83	−0.27
SAR	**0.97**	−0.18	−0.14	EC	0.29	0.10	0.88
ESP	**0.97**	−0.18	−0.14	Ex. Ca	−0.87	0.00	0.10

The values in bold represent essential contributions that are above the expected value if the contributions were uniform.

## Data Availability

The supporting data for the study are all provided in the publication, and if you require any additional data, you may contact the corresponding author for further assistance.
